# Early development of attention to threat-related facial expressions

**DOI:** 10.1371/journal.pone.0197424

**Published:** 2018-05-16

**Authors:** Jukka M. Leppänen, Julia K. Cataldo, Michelle Bosquet Enlow, Charles A. Nelson

**Affiliations:** 1 Infant Cognition Laboratory, Center for Child Health Research, Faculty of Medicine and Life Sciences, University of Tampere, Tampere, Finland; 2 Division of Developmental Medicine, Boston Children's Hospital, Harvard Medical School, Boston, Massachusetts, United States of America; 3 Department of Psychiatry, Boston Children's Hospital, Harvard Medical School, Boston, Massachusetts, United States of America; 4 Harvard Graduate School of Education, Harvard University, Boston, Massachusetts, United States of America; University of Melbourne, AUSTRALIA

## Abstract

Infants from an early age have a bias to attend more to faces than non-faces and after 5 months are particularly attentive to fearful faces. We examined the specificity of this “fear bias” in 5-, 7-, and 12-month-old infants (*N* = 269) and 36-month-old children (*N* = 191) and whether its development is associated with features of the early rearing environment, specifically maternal anxiety and depression symptoms. Attention dwell times were assessed by measuring the latencies of gaze shifts from a stimulus at fixation to a new stimulus in the visual periphery. In infancy, dwell times were shorter for non-face control stimuli vs. happy faces at all ages, and happy vs. fearful, but not angry, faces at 7 and 12 months. At 36 months, dwell times were shorter for non-faces and happy faces compared to fearful and angry faces. Individual variations in attention dwell times were not associated with mothers’ self-reported depression or anxiety symptoms at either age. The results suggest that sensitivity to fearful faces precedes a more general bias for threat-alerting stimuli in early development. We did not find evidence that the initial manifestation of these biases is related to moderate variations in maternal depression or anxiety symptoms.

## Introduction

Human brain development is dependent on access to optimal environments, with adequate levels of sensory and social stimulation [1, 2]. For example, the formation of ocular dominance columns in the primary visual cortex [3] and the associated onset of binocular visual function [4] are closely linked to the duration of postnatal visual experience. Similar experience-driven neural changes may occur in higher-level visual systems involved in face processing [5], [6]. Here, an early-emerging and coarse component, such as newborns’ ability to orient to face-like patterns [7] or to faces that appear to look at the infant [8], is refined over time and becomes more selectively tuned to faces that are most common in the infant’s environment. For example, the tendency to “prioritize” attention to faces over other visual objects in monkeys is broadly tuned to human and monkey faces at birth but becomes more selective to faces that are more common in the rearing environment, be they human or monkey faces [6]. Possibly reflecting similar experience-dependent changes in humans, infants’ attentional bias for faces becomes stronger between 6 and 12 months of age [9–11] as well as more selective to faces of one’s own race [12]. Children may also become increasingly attuned to facial expressions that are relatively more frequent in the environment (e.g., physically abused children exhibit enhanced attention to angry faces) [13].

As an experience-dependent capacity, the development of face processing in infants may be subject to individual variations, arising from differences in various aspects of the early environment. While a powerful test of this hypothesis would be provided by studies of children exposed to drastic reductions in access to social interaction (e.g., due to institutionalized care or parental neglect [2, 14]), the development of attention to faces may also be sensitive to more ordinary variations in infant-parent interactions (e.g., changes arising from parental anxiety or depression symptoms. Parental mood symptoms have been associated with observable differences in parents’ (i.e., mothers’ and/or fathers’) behavior, including reduced parental facial expressions during interaction [15], reduced attention to infant cues and emotion expressions [16], and altered acoustic characteristics of speech directed toward the infant [17]. The nature of these variations may vary, depending on whether the parent is experiencing primarily depression vs. anxiety-related symptoms. For example, a study that examined brief periods of naturalistic interactions between infants and parents found relatively reduced positive affect expressions in dyads with lifetime history of parental depressive symptoms, and increased duration of infant gaze to parent as well as increased expressions of positive and negative emotions in dyads with an anxious parent [15]. These experiences may help shape the development of infants’ face processing systems above and beyond any influence of shared genetic liability for specific behavioral traits.

In the current study, we examined attention to happy, fearful, and angry facial expressions in infancy (i.e., at 5, 7 or 12 months) and early childhood (36 months). We also examined whether individual variations in attention to facial expressions, particularly threat-alerting cues in the form of fearful and angry expressions, are associated with variations in maternal anxiety and depression symptoms. Specifically, we assessed infants’ attentional dwell times to happy, fearful, and angry facial expressions, as well as for a non-face pattern, while a salient competing stimulus was shown in the visual periphery. Several studies using this approach in 5- to 36-month-old children have shown that dwell times are longer for faces as compared to non-face patterns [11, 18–21]. Between 5 and 7 months, this tendency to maintain gaze on faces becomes sensitive to facial expression. For example, whereas 5-month-old infants do not yet differentiate between static fearful and happy faces [11, 22] (although also see [23], 7- and 9-month-old infants show consistently longer dwell times for fearful as compared to neutral and happy faces [11, 20, 21, 24]. It is not known whether the difference in dwell times for fearful vs. happy faces reflects an early-emerging sensitivity to some aspects of fearful or “gasping” faces [25], or is a developmental precursor of a more general bias to “threat-alerting” stimuli [26]. Results from previous studies suggest that the dwell-time bias for fear may not be found for angry faces in 8- to 14-month-old [27] or 4- to 24-month-old [28] infants; however, systematic comparisons of infants’ dwell times for fearful and angry expressions within the same study and across sufficiently large groups of infants from different age levels have not been conducted.

Based on previous studies, we hypothesized that infants will maintain gaze longer on faces vs. non-face patterns across all ages, and fearful vs. happy faces starting at age 7 months. Extending previous studies, we examined whether the bias for fearful vs. happy expressions in 7-month-old and older infants generalizes to other negative emotions (e.g., anger). A limited number of studies have examined associations between infants’ attention biases and maternal mood symptoms. These studies suggest that attention to faces in general is positively correlated with maternal anxiety symptoms [15, 29] and that attention to facial expressions of fear or anger is selectively enhanced in the context of parental stress and depression/anxiety symptoms [28, 30]. By including data from 5- to 12-month-old infants and 36-month-old children, we were also able to explore age effects. Age-dependent effects may be expected if the underlying processes and their sensitivity to the environment are transient (e.g., confined to the peak of the preference for faces in infancy) *or* if the developmental time-course of face processing is affected by the select features of the environment (e.g., some aspects of attention to faces emerge earlier or later in certain environments).

## Method

### Participants and design

The current analyses use data from a longitudinal cohort study on the development of emotion processing during the first years of life. The study was conducted at Boston Children’s Hospital/Harvard Medical School. Families were enrolled when the children were 5, 7, or 12 months old (laboratory visit, questionnaires) and were then followed when the child was 24 months (questionnaires) and 36 months (laboratory visit, questionnaires). Families with children in the target age range were recruited from a participant registry that had been established in the Laboratory in 2005, and now contains the names of nearly 30,000 individuals who have expressed interest in participating in research. Parents provided written informed consent before the start of the study. Ethical permission for the study was obtained from the Institutional Review Board of Boston Children’s Hospital.

The current analyses were started after all infant assessments (5, 7, or 12 months) for the longitudinal study were completed. All available data were used with the exception of the data of 21 participants who met a priori exclusion criteria for all analyses (i.e., prenatal exposure to antipsychotic, anticonvulsant, or narcotics medications, genetic abnormalities, or a developmental delay). Data were available for a total of 642 children for the 5- to 12-month age groups. Of these, 269 had completed the 36-month follow-up assessment by the time of the current analyses. The final analyses used data from participants with successful calibration of >3 points in the eye tracking calibration and >2 valid trials per experimental condition. The percentage of children for each age group who met the a priori criteria for inclusion in the final analyses of eye-tracking data are reported in Table 1. The sample size in the final analyses linking eye-tracking results with measures of maternal anxiety/depression symptoms in infancy was 269 for infants and 191 for 36-month-old children, giving 80% power to detect associations that varied from .21 (infants) to .25 (children) at a corrected alpha of .008. Children in the final sample were White (78.1%), Asian (3.0%), African American (1.9%), Asian Indian (0.4%), Pacific Islander (0.4%), multi-racial (14.8%), or of unreported (1.5%) racial origin. The majority of mothers had completed higher education, including a Bachelor’s (31.3%), Master’s (42.5%) or a Doctoral (20.9%) degree; total annual household income was less than $50,000 (7.3%), between $50,000 and $74,999 (10.6%), $75,000 and $99,999 (15.9%), or $100,000 or greater (65.0%). Participants who met the inclusion criteria for the current analyses did not differ from those who did not meet the inclusion criteria with respect to either maternal anxiety or depression symptoms, all *p*s > .05.

**Table 1:** Number of children (N_final_) included, having met successful calibration and sufficient number of valid trials, by age group.

Age (m)	*N*_original_	Calib.	Trial	*N*_final_ (%)	Female (%)	Age in days (range)
5	177	40	75	62 (34%)	29 (47%)	152 (135–161)
7	197	23	63	111 (56%)	41 (38%)	212 (205–223)
12	268	45	121	102 (37%)	47 (46%)	365 (354–374)
36	269	19	49	201 (75%)	77 (43%)	1157 (1089–1344)

### Eye-tracking assessments

During the laboratory visit, participants were assessed with a battery of tests, including measurements of baseline EEG/event-related potentials (ERPs) or functional Near-Infrared-Spectroscopy (fNIRS) responses to pictures of facial expressions, and eye-tracking measurement of attentional dwell times for non-face control stimuli as well as faces displaying happy, fearful, and angry expressions. Data from the EEG, ERP and fNIRS tasks will be reported separately. The eye-tracking test was implemented after the EEG/ERP or fNIRS task.

Eye-tracking assessment took place in a sound-attenuated and dimly lit room. During the infant assessment, the infant was seated on his/her parent’s lap at a ~65cm viewing distance in front of a 19-inch computer monitor equipped with a Tobii T120 eye-tracker (Tobii Technology, Stockholm, Sweden). The eye-tracking session was started by a calibration of the eye-tracking cameras, using the standard calibration procedure within the Tobii Studio software. During the calibration procedure, a red dot was presented against a grey background in five locations, including the center of the screen and the four corners of the screen. The outcome of the calibration was assessed from a plot showing error vector for the five calibration points. If one or more of the five calibrations points were missing or were not properly calibrated (i.e., had large error vectors), the calibration for the missing locations was retried. If calibration points were missing after two recalibration attempts, the final calibration outcome was accepted, and the test started.

Following eye tracker calibration, a test programmed to E-Prime 2.0 software (Psychology Software Tools, Pittsburgh, PA) was administered to assess infants’ attention to non-face control stimuli and faces [11, 18–21, 31]. Each trial started with a dynamic attention-grabbing stimulus presented on the center of the screen. After the infant fixated on the stimulus, as judged by the experimenter monitoring the infant via a video camera, two test stimuli were presented. The first was presented on the center of the screen for 4000 ms. The second stimulus was presented with a 1000-ms onset asynchrony laterally on the left or right side of the screen with 13.6° eccentricity, and remained on the screen for 3000 ms. From the viewing distance of 65 cm, the first stimulus measured 14.3° and 11.2° vertically and horizontally and was a picture of a non-face pattern or a picture of a face displaying a happy, angry, or fearful expression. The non-face patterns were created by randomizing the phase spectrum of the face stimuli, and by cropping the resulting image to the outline of a face (following [21, 32]). The face stimuli were pictures of two female models (models # 5 & 8), selected from the NimStim stimulus set [33]. Infants saw pictures of only one of the two models, but the model used was counterbalanced across participants. The second stimulus (13.0° x 3.5°) was a geometric shape (vertically arranged black and white circles or a checkerboard pattern). Infants were presented with 6 trials per experimental condition. Testing was paused if the infant became fussy and terminated if continuing the testing would have been too distressing for the infant.

The eye-tracking test used in the 36-month assessment was similar with the exception that the onset asynchrony of the first (central) and the second (lateral) stimulus was reduced from 1000 ms to 200 ms, and the content of the peripheral stimuli were changed from black-and-white patterns to colourful patterns. These changes were undertaken to render the task more attractive for this age group. In addition, the child was not seated on his/her parent’s lap during the task.

Trial data, including timestamps (Tobii TETtime) corresponding to the onset times of central and peripheral pictures, and xy coordinates of the participants’ eyes and their respective validity estimates, as given by Tobii, were stored in Tobii gazedata output files. All analyses of saccadic eye movements from the central stimulus to the lateral stimulus were implemented offline using an automated MATLAB script and criteria described in prior studies [34]. Briefly, trials with (a) a sufficient fixation on the central stimulus (i.e., >70% of the time) during the time preceding gaze shift or the end of the analysis period (i.e., 1000 ms after the lateral stimulus onset), (b) sufficient number of valid samples in the gaze data (i.e., no gaps longer than 200 ms), and (c) valid information about the eye movement from the central to the lateral stimulus (i.e., the eye movement did not occur during a period of missing gaze data) were retained for analysis. The duration of attention dwell time on the first stimulus (face or non-face pattern) was determined for the period starting 150 ms from the onset of the lateral stimulus and ending 1000 ms after the lateral stimulus onset, and converted to a normalized dwell time index score by using the following formula:

$$Dwell\;time\;index=\frac{\sum_{i=1}^{n}\left(1-\frac{1000-x_{i}}{850}\right)}{n}.$$

In this formula, *x* is the time point of the saccadic eye movement (i.e., the last time point when gaze is in the area of the first stimulus preceding a saccade towards the lateral stimulus), and *n* is the number of scorable trials in a given stimulus condition [34]. The shortest acceptable saccadic eye movement latency (150 ms) results in an index value of 0, and the longest possible latency (or a lack of saccade, which is equal to the last measured time point of the first stimulus at 1000 ms) in an index value of 1. Dwell time indices were calculated separately for each of the four stimulus conditions (i.e., non-face, happy, angry, fearful). It is noteworthy that the current method of calculating dwell time indices is comparable to the more commonly used approach for calculating mean saccadic latency or saccadic reaction time measures with the exception that the current approach does not exclude trials without gaze shift (or reaction times censored at the 1000 ms cut-off). The current approach is preferable given that the probability of trials without gaze shifts can be relatively high in developing populations [34].

### Maternal symptomatology questionnaires

Prior to each laboratory visit, the child’s caregiver completed a battery of questionnaires via an online survey. The current analyses include self-reports of anxiety symptoms, assessed via the trait subscale of the State-Trait Anxiety Inventory (STAI, [35]), and depression symptoms, assessed via the Beck Depression Inventory (BDI-II, [36]). Caregivers were also asked about the use anxiolytic and antidepressant medications; given the low percentage of reported use for these medications (i.e., 0.6% and 5.6%, respectively), current analyses were not separated by reported medication use. For most participants, the questionnaires were filled out by the biological mother, except for a few questionnaires that were filled out by the child’s father (*n* = 8). Given the focus of the current study on maternal anxiety/depression, symptom questionnaires filled out by fathers were not used in the analyses (*n* = 3 to 8, depending on analyses).

The STAI trait subscale was designed to assess normative variations in propensity to perceive various situations as threatening and to experience anxiety [35]. The respondents were asked to evaluate a total of 20 statements (e.g., “I worry too much over something that really doesn’t matter”) for frequency on a four-point scale (i.e., “almost never”, “sometimes”, “often,” and “almost always.”), resulting in a total score that could range from 20 to 80. For the current analyses, the total score was calculated by averaging individual responses and multiplying the average by 20 to account for the impact of rare missing values on the total score. Internal consistency estimates have been reported from .86 to .95 [35].

The BDI-II includes 21 sets of statements that relate to characteristic attitudes and symptoms of depression and vary in severity (e.g., “I feel sad” vs. “I am so sad and unhappy that I can’t stand it”). The statements from each set were scored from 0 to 3, resulting in a total score that could range from 0 to 63. For the current analysis, the average item score was calculated and multiplied by 21 to obtain a total depression symptom score. Internal consistency estimates have been reported from .73 to .92 [37].

### Statistical analyses

Given that many of the study variables were not normally distributed, we used non-parametric tests for analysing differences between two or more age groups (i.e., Mann-Whitney U or Kruskal-Wallis H tests), within-subject variation between stimulus conditions (i.e., Friedman tests or Wilcoxon tests), and partial correlations (Spearman rho, http://imaging.mrc-cbu.cam.ac.uk/statswiki/FAQ/partsp)). The analyses were implemented using SPSS statistical analysis package, version 23.

In descriptive analyses, we compared the number of valid trials in each age group and stimulus condition. We also estimated measurement error by calculating odd-even split-half correlations. To examine differences in attention dwell times between stimulus conditions, we compared dwell times for non-face patterns vs. happy faces (to assess bias towards non-threatening faces), happy vs. fearful expressions (to assess bias towards fearful expressions), and happy vs. angry faces (to assess bias towards angry expressions). The tests were evaluated against a Bonferroni-adjusted alpha of .017. To compare differences between ages, we created “bias” scores for happy faces (Dwell_Happy_/Dwell_Non-Face Patterns_), fearful faces (Dwell_Fearful_/Dwell_Happy_), and angry faces (Dwell_Angry_/Dwell_Happy_), and tested whether these scores differed between the three age groups (corrected alpha = .017). Data from the 36-month assessment were analysed separately.

To test the hypotheses regarding the associations between dwell times and maternal anxiety/depression symptoms, separate sets of analyses examining associations between dwell times for happy, fearful, and angry faces with measures of maternal anxiety or depression were conducted. Dwell times for non-face stimuli were used as a control variable in analyses linking dwell time for happy faces with maternal anxiety/depression, and dwell times for happy faces were used as a control variable in analyses linking dwell times for fear and anger with maternal anxiety/depression. Again, data from the 5- to 12-month and 36-month visits were analysed separately. For both analyses, reports of maternal anxiety/depression obtained in infancy (i.e., at 5–12-month visit) were used as measures of the early emotional environment; for the analyses of the 36-month data, anxiety/depression ratings obtained at the 36-month assessment were also considered. The results of these analyses were evaluated against a corrected alpha of .008.

## Results

### Descriptive statistics for eye-tracking measures

The percentage of valid trials and mean dwell times in each age group and stimulus condition are shown in Table 2. There were no significant differences in the number of valid trials between age groups in any of the stimulus conditions or between stimulus conditions (all *p*s > .0125). The number of valid trials was not significantly associated with dwell times in any of the stimulus conditions in infancy or at 36 months of age, all *p*s > .0125. The odd-even split-half correlations (Spearman rho) ranged from .31 to .44 (Mean = .36) in infancy and between .41 and .49 (Mean = .46) at 36 months, all *p*s < .001. Correlations in dwell times across stimulus categories (i.e., between non-face control stimuli and different face conditions) were .35-.41 (Mean = .38) in infancy and .35–38 (Mean = .36) at 36 months, whereas within-category correlations (i.e., between facial expressions) were .57-.63 (Mean = .60) in infancy and .64-.70 (Mean = .67) at 36 months, all *p*s < .001.

**Table 2:** Number of valid test trials and mean values of dwell times for each age group and stimulus condition.

	**Valid test trials**			
**Age (months)**	**Non-Face**	**Happy**	**Angry**	**Fearful**
**5**	4.7 (1.0)	5.0 (1.0)	5.0 (1.1)	5.2 (0.9)
**7**	4.9 (1.0)	4.8 (1.0)	5.0 (1.0)	5.0 (1.0)
**12**	4.7 (1.0)	4.7 (1.0)	4.6 (1.0)	4.8 (1.0)
**5−12**^**1**^	4.8 (1.0)	4.8 (1.0)	4.8 (1.0)	4.9 (1.0)
**36**	5.0 (1.0)	5.0 (0.9)	5.0 (1.0)	5.0 (1.0)
	**Mean dwell times (SD)**			
**Age (months)**	Non-Face	Happy	Angry	Fearful
**5**	.32 (.19)	.41 (.25)	.42 (.22)	.41 (.23)
**7**	.31 (.17)	.41 (.22)	.40 (.21)	.45 (.23)
**12**	.35 (.21)	.44 (.23)	.48 (.25)	.53 (.25)
**5−12**^**1**^	.33 (.19)	.42 (.23)	.44 (.23)	.47 (.24)
**36**	.55 (.20)	.54 (.25)	.62 (.22)	.62 (.22)

Our supplementary analyses showed that the experimental conditions preceding the eye-tracking tests affected dwell times in infants and children (see S3 File). To control for these effects, the scores were mean-centered within each condition for all analyses examining associations between dwell time variables and maternal anxiety/depression scores. Two-sample Kolmogorov-Smirnow tests showed no differences between groups after mean centering, indicating that, while the preceding condition affected the location of the dwell-time distributions (i.e., infants/children with less exposure to faces preceding the eye-tracking session tended to have longer dwell times in the eye-tracking test), it had no effect on the distribution of individual dwell times within conditions.

### Descriptive statistics for maternal anxiety and depression symptoms

Descriptive statistics for maternal anxiety and depression symptoms are reported in Table 3. There were no differences between age groups on any of the maternal variables. There was a significant positive correlation between anxiety and depression symptoms in infancy, Spearman rho = .59, *p* < .001, and at 36 months, Spearman rho = .62, *p* < .001. Also, anxiety and depression symptom scores were moderately stable between infancy and 36 months, Spearman rho = .70 and .37, respectively, *ps* < .001.

**Table 3:** Descriptive data for maternal anxiety (STAI) and depression (BDI) symptoms.

	Min	Max	Mean (SD)	Skewness	Kurtosis
**STAI, 5–12 months**	20.0	64.0	34.6 (8.3)	0.7	0.2
**STAI, 36 months**	21.0	59.0	33.0 (7.6)	0.9	0.8
**BDI, 5–12 months**	0.0	27.0	5.6 (4.4)	1.5	3.7
**BDI, 36 months**	0.0	39.5	5.4 (4.9)	2.7	13.5

### Differences in dwell times between stimulus conditions and across ages

**Fig 1:**
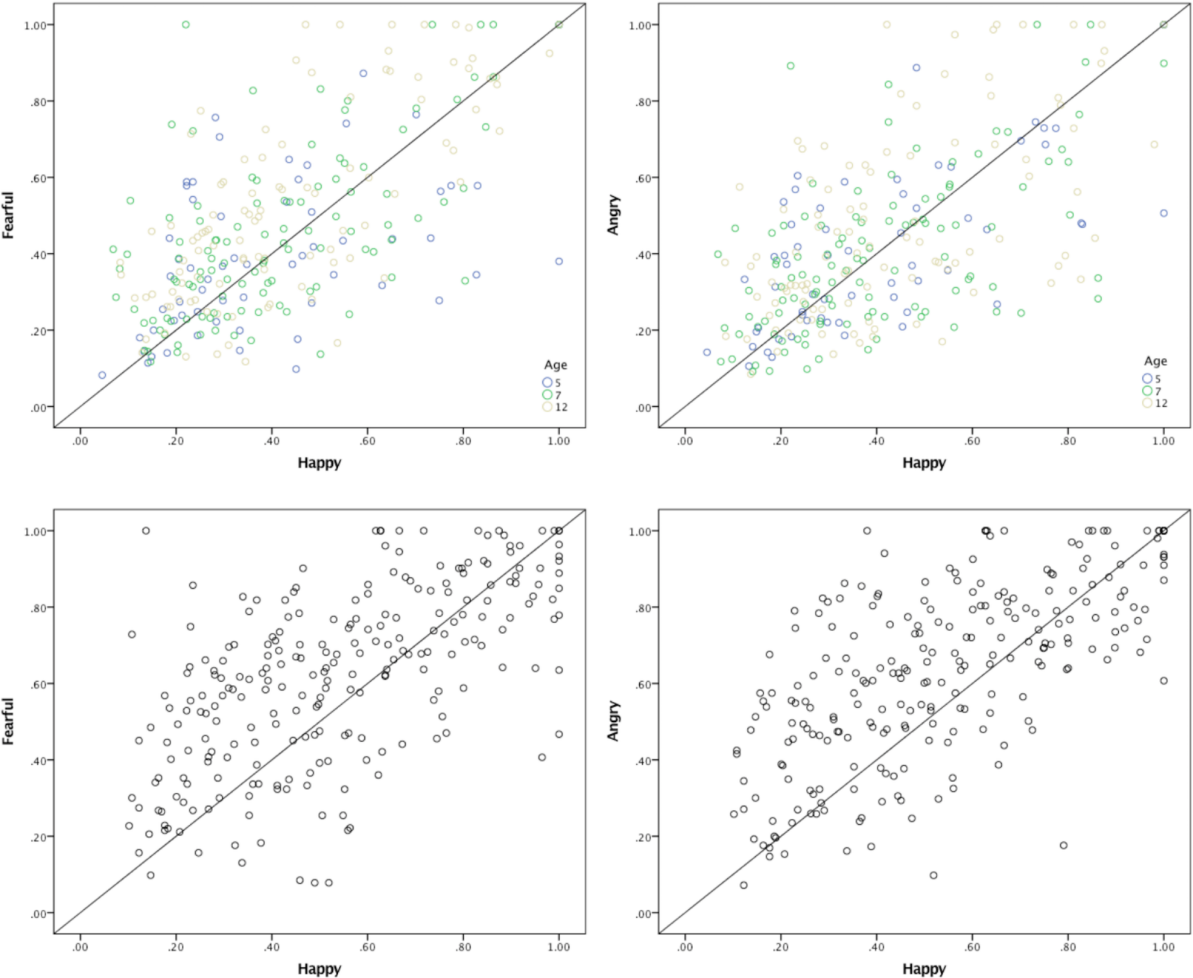
Attention dwell times at 5 to 12 months (top) and 36 months (bottom) of age. The scatterplots show dwell times from individual participants for happy vs. fearful faces (left) and happy vs. angry faces (right). Values above the grey diagonal lines indicate a bias for fearful (left) or angry (right) faces.

In a combined analysis of data from all infant age groups, dwell times were shorter for non-face patterns compared to happy faces, *Z* = 6.2, *p* < .001, as well as happy compared to fearful faces, *Z* = 4.5, *p* = .001. There was no difference in dwell times for happy vs. angry faces, *p* > .05 (Fig 1). There were no significant age-related differences in infancy in the attention bias scores for facial expressions, with the exception of a marginal effect of age on the bias for fear, χ2 = 6.3, *p* = 0.04 (corrected alpha = .017). This effect reflected the fact that the difference in dwell times for happy vs. fearful faces was not found at 5 months, was marginal in 7-month-olds, Z = 2.4, *p* = .018, and significant in 12-month-olds, Z = 4.6, *p* < .001.

At 36 months, the dwell times were not different for the non-face pattern vs. happy faces, but significant differences were found between happy vs. fearful faces, *Z* = 5.8, *p* < .001, and happy vs. angry faces, Z = 6.6, *p* < .001.

### Associations between child attention and maternal anxiety/depression symptoms

Hypotheses regarding the associations between dwell times and maternal anxiety/depression symptoms were tested by examining partial correlations between dwell time variables and measures of maternal anxiety and depression symptoms. Dwell times for non-faces were controlled to examine whether attention to happy faces correlates with maternal symptomatology, and dwell times for happy faces were controlled to examine whether biases for fearful or angry faces correlate with maternal symptomatology. In a combined analysis of data from the 5-, 7-, and 12-month-old infants, the biases for happy, fearful, and angry faces were not associated with maternal anxiety or depression symptoms. The results did not change when the analysis was confined to the 7- and 12-month age groups (i.e., the two groups that exhibited a bias in attention dwell times for fearful expressions). Among the 36-month-olds, neither the biases for happy faces nor for fear or anger were significantly associated with either maternal anxiety or depression symptoms (all correlation coefficients < .11).

## Discussion

The present study yielded three main findings. First, consistent with prior studies [11, 20], our results showed shorter dwell times for non-face stimuli vs. happy faces across all ages in infancy, and shorter dwell times for happy vs. fearful faces at 7 and 12 months of age. Second, the bias for fear did not generalize to expressions of anger in infancy but biased looking to both fearful and angry faces was found at 36 months. Third, the analyses showed that the dwell time biases for happy, fearful, and angry faces were not significantly associated with maternal anxiety or depression symptoms.

Our results concur with previous studies in showing differential attentional patterns for fearful and angry expressions in infants. In previous studies, it has been found that whereas 8- to 14-month-old infants look longer at fearful than at happy expressions, they show no difference in looking times for happy vs. angry expressions when pictures depicting these two expressions are presented side by side ([27], see also [28]). Our study adds to these findings by showing that the well-documented delay in disengaging from fearful expressions is not found for angry expressions in a large and well-powered sample of 7- to 12-month-old infants. Hence, our results suggest that the bias to look at fearful expressions in infants may not arise from a general bias towards “threat-alerting” stimuli (both fearful and angry expressions are associated with threat) or negatively valenced expressions [26]. It is possible, therefore, that infants’ attention is captured by some unique physical characteristics of fearful expressions (e.g., wide-open eyes, although see [38] or the novelty of this facial expression in typical rearing environments [39], (although see 21). Infants’ attention to fearful faces may also reflect an example of adaptive perceptual attunement to signals that warn about potential danger in the environment [40, 41], although there is currently no direct evidence for this kind of “rich” interpretation.

Whereas there were no indications for an attention bias towards negative (i.e., fearful and angry) expressions in the infancy assessments, a clear difference in dwell times for happy vs. fearful *and* angry expressions was found at 36 months. These age-related differences in relative weighting of attention to different stimuli points to developmental changes in attention to facial expressions over early childhood and suggest that the bias for fear precedes a more general bias for threat-alerting cues in early childhood.

Our results further showed that the magnitudes of the attention bias towards happy, fearful, and angry faces were not associated with variations in mothers’ self-reported anxiety or depression symptoms. This result is in contrast with previous studies showing that attentional bias towards fearful vs. happy faces [30] and angry vs. happy faces [28] is more pronounced in infants and young children whose mothers reported elevated depression or anxiety symptoms. The current study is comparable to these previous studies with respect to methods used to assess infants’ attention biases as well as the distribution of depression and anxiety symptoms in the sample, but the studies differed in the questionnaires that were used to assess depression and anxiety symptoms. It is therefore possible that the association between infants’ early emerging attentional biases and maternal mood symptoms is not robust across different methodological approaches.

A number of limitations might have affected the power of the association analyses in the current study. The current sample consisted of primarily healthy, high socioeconomic status individuals with a restricted range of variability in anxiety/depression symptoms, and assessments of mood symptoms were limited to the infants’ primary caregiver. Further, given that the infants were tested with a rather lengthy EEG/fNIRS paradigm before the eye-tracking tests (S3 File), the retention rate in the current analysis was only 35 to 54%, which is noticeably lower than the 82 to 100% in previous infant eye-tracking studies using similar paradigms [34, 42, 43]. Collectively, these considerations raise questions about the representativeness of the sample, particularly with regards to the variables reflecting features of the rearing environment. These concerns are, in part, alleviated by our additional analyses showing that infants who met the inclusion criteria for the current analyses did not differ from those who did not with respect to maternal anxiety and depression symptoms. Also, our additional analyses showed no associations between test success rates, as assessed by the total number of valid trials in the analyses, and infants’ dwell times or caregiver anxiety/depression symptoms. Hence, while we acknowledge that the low retention rate is a limitation in the current analyses, there are no indications that this has affected the representativeness of the sample with respect to the key study variables.

An additional limitation of the current association analyses concerns measurement noise. Estimates of the split-half reliability varied across stimulus conditions, and only some of them were within the range reported in previous studies in infants [44]. It is noteworthy that, whereas the reliability estimates in the current analyses were computed to compare our measures with those in previous studies with infants, they are likely to underestimate the true reliability of the scores, given attenuation due to low number of trials in the odd-even split-half analysis. Applying the Spearman-Brown correction for the reliability estimates [45], the mean true reliability of the individual scores is likely to be .50 for dwell times in individual conditions. Thus, while it is clear that measurement error may have attenuated associations in the current analyses, it appears unlikely that measurement error led to a complete masking of an association between attention dwell times and maternal symptoms. The observed associations were low (< .11) in the current analyses, and even if these correlations are adjusted for measurement error by dividing the correlation by the geometric mean of the estimated reliabilities of the two measures being correlated [45, 46], the correlation coefficients remain low.

In conclusion, the results of this study replicated previous studies in showing a clear attentional preference for faces in 5- to 12-month-old infants and a preference for fearful over happy expressions in 7-, 12- and 36-month-old children. As an important extension of previous studies, the current results further indicated that the bias for fear in infants cannot be interpreted as a more generalized bias for expressions of negative emotion, as the bias was not found for angry facial expressions. It remains possible, however, that the more generalized bias for different negative emotions emerges later in development, as suggested by our findings showing differences in dwell times for happy vs. fearful *and* vs. angry expressions in 36-month-old children. Contrary to some previous results [28, 30], the current findings showed that individual differences in the early biases for faces and fear were not associated with maternal mood, raising the possibility that these biases emerge relatively independently of normative variations in the rearing environment. However, further research is needed to replicate these results in more heterogeneous samples with greater variability in parental mood symptoms.

## Supporting information

S1 File Trial-by-trial dwell time data and questionnaire data. Missing values are marked as “#NULL!”. Variable names, types, labels, and value labels are explained in the variable codebook–document (S2 File).
(XLSX)

S2 File Variable codebook. (PDF)

S3 File Supplementary analyses examining the effects of participant gender and the preceding experimental condition on eye tracking results. (PDF)
