# Supplementary material for: Early development of attention to threat-related facial expressions
Source: PLoS One. 2018 May 16;13(5):e0197424. doi: 10.1371/journal.pone.0197424 (PMC5955579; doi:10.1371/journal.pone.0197424)
Supplement: S2 File — (PDF) [file pone.0197424.s002.pdf]

Variables in the "Early Development - Eye Tracking and Questionnaire Data" dataset

Variable	Label	Measurement Level
id	Participant ID	Scale
Trialnumber	Trial number	Scale
CentralStimulus	Stimulus Condition (Non-Face, Happy, Fearful, Angry)	Nominal
LateralStimSide	Location of the lateral stimulus (Left, Right)	Nominal
FaceTime	Duration of the first part of the trial (Face) in milliseconds	Scale
LateralStimTime	Duration of the second part of the trial (Face + Lateral Stimulus) in ms	Scale
AnalysisPeriodLength	Length of the analysis period	Scale
LastTimeInCenterAOI	Last time gaze recorded in the central area	Scale
FirstTimeInLateralAOI	First time gaze recorded in the lateral area	Scale
ValidGazeR	% Valid gaze, right eye	Scale
ValidGazeL	% Valid gaze, left eye	Scale
LongestNonValidStreakR	Duration of the longest non-valid data streak (right eye)	Scale
LongestNonValidStreakL	Duration of the longest non-valid data streak (left eye)	Scale
LongestNonValidStreakCombined	Duration of the longest non-valid data streak (both eyes)	Scale
InsideCenteralAOIBefGazeShift	% of gaze inside central AOI before gaze shift to the lateral stimulus/end of the analysis period	Scale
AOIBorderViolationYesNo	Gaze shift between AOIs during a period of non-valid data	Nominal
SRT	Saccadic Reaction Time (-1, missing value)	Scale
DwellIndex	Normalized dwell time index score (-1, missing value)	Scale
AgeGroup	Age group in months (5, 7, 12, or 36)	Nominal
Model	Face model	Nominal
OddTrial	1 = Odd numbered trial, 0= even numbered trials	Nominal
Sex	Sex of the infant	Nominal
Race	Child's race	Nominal
STAI_Trait_Mx20_Inf	Composite trait anxiety from the Spielberger State and Trait Anxiety Inventory (STAI; Mean * 20 - handling missing values) (Infant visit)	Scale
BDI_Mx21_Inf	Total depression score from the Beck Depression Inventory (BDI; Mean * 21 - handling missing values) (Infant visit)	Scale
STAI_Trait_Mx20_3yr	Composite trait anxiety from the Spielberger State and Trait Anxiety Inventory (STAI; Mean * 20 - handling missing values) (3yr visit)	Scale

BDI_Mx21_3yr	Total depression score from the Beck Depression Inventory (BDI; Mean * 21 - handling missing values) (3yr visit)	Scale
P1_Education	Parent 1 highest level of education	Nominal
Family_Income	Total combined family income over the past 12 months	Nominal
PrecedingExpCondition	Experimental condition preceding the eye tracking test	Nominal
PrecedingStudyType	Neuroimaging method used in the experiment preceding experimental condition	Nominal
calib_qual_inf	Quality of eye-tracking calibration (infant visit)	Scale
calib_qual_3yf	Quality of eye-tracking calibration (3-year visit)	Scale
Respondent_Inf_STAI_BDI	Relationship of the person who filled out the questionnaires to the infant	Nominal
Respondent_3yr_STAI_BDI	Relation of the person who filled out the 3yr questionnaires to the infant	Nominal
AgeInfantVis	Age in days (infant visit)	Scale
Age3yVis	Age in days (3y visit)	Scale

Variable Values

Value		Label
OddTrial		1 = Odd numbered trial, 0= even numbered trials
Sex	0	Male
	1	Female
Ethnicity	0	Non Hispanic, Latino/a, or Spanish origin
	1	Mexican, Mexican American, or Chicano/a
	2	Puerto Rican
	3	Cuban
	4	Other Hispanic, Latino/a, or Spanish origin
	5	Mixed Hispanic, Latino/a, or Spanish origin
	9	Did not respond
Race	0	White
	1	Black or African American
	2	American Indian or Alaska Native
	3	Asian Indian

	4	Asian (Chinese, Japanese, Korean, Vietnamese, or Other)
	5	Pacific Islander (Filipino, Native Hawaiian, Guamanian or Chomorro, Samoan, or Other)
	6	Mixed Race
	9	Did not respond
P1_Education	1	8th Grade or Less
	2	Some High School
	3	High School/GED
	4	Associates Degree
	5	Bachelors Degree
	6	Masters Degree
	7	M.D., Ph.D., J.D. or Equivalent
Family_Income	0	Don’t Know
	1	Less than $5,000
	2	$5,000 through $11,999
	3	$12,000 through $15,999
	4	$16,000 through $24,999
	5	$25,000 through $34,999
	6	$35,000 through $49,999
	7	$50,000 through $74,999
	8	$75,000 through $99,999
	9	$100,000 and greater
PrecedingExpCondition	1	Human Faces
	2	Animal Faces
	99	N/A
PrecedingStudyType	1	ERP
	2	NIRS
Respondent_Inf_STAI_BDI_Cleaned	1	Mother
	2	Father
	3	Other - See Respond_Specify
Respondent_3yr_STAI_BDI_Cleaned	1	Mother
	2	Father
	3	Other
