# Supplementary material for: Early development of attention to threat-related facial expressions
Source: PLoS One. 2018 May 16;13(5):e0197424. doi: 10.1371/journal.pone.0197424 (PMC5955579; doi:10.1371/journal.pone.0197424)
Supplement: S3 File — (PDF) [file pone.0197424.s003.pdf]

S3. Supplementary data analyses for ”Early Development of Attention to Threat-Related Facial Expressions”

Supplementary analyses were conducted to examine whether participant gender and the experimental condition preceding the eye tracking test session had any effect on eye tracking results.

There was no effect of gender on dwell times in any of the conditions in infancy or at 36 months, all *p*s $> .10$. Given that the physiological test preceding the eye tracking task varied across participants in the current sample (i.e., the infants were presented with either animal or human faces while EEG or NIRS activity was recorded), we also examined whether the preceding experimental condition had any effect on infants' eye tracking measures. This analysis revealed a significant effect of the preceding experimental condition so that the sub-group presented with animal faces during the EEG/NIRS recording (n= 45) had longer dwell times in all face conditions as compared to the group presented with human faces during the EEG/NIRS recording, *Z*s >3.7, *p*s $< .003$. Similar analyses were not conducted for the 36-month data because the animal face condition was not continued in the follow-up assessments. There was no effect of neuroimaging method on the dwell times (i.e., whether the infant was tested by the EEG or fNIRS paradigm prior to the eye tracking session), although infants who were tested with the fNIRS procedure before the eye tracking session tended to have slightly longer dwell times in all conditions than infants in the EEG group, *Z*s 0.8-1.8, *p*s = .08-.45. These differences were significant for all face conditions in the 36-month assessment, Mann-Whitney *Z*s >6.4, *p*s $< .001$. These condition effects are likely to be explained by the differences in the amount of exposure to faces in the animal vs. human face conditions, or fNIRS vs EEG sessions, and differential

habituation levels to faces at the start of the eye tracking test session (i.e., infants/children with less exposure to faces preceding the eye tracking session tended to have longer dwell times in the eye tracking test).
